# Draft Genome Sequence of Insecticidal *Streptomyces* sp. Strain PCS3-D2, Isolated from Mangrove Soil in Philippines

**DOI:** 10.1128/genomeA.00448-14

**Published:** 2014-06-12

**Authors:** Aileen N. Bayot-Custodio, Edwin P. Alcantara, Teofila O. Zulaybar

**Affiliations:** National Institute of Molecular Biology and Biotechnology (BIOTECH), University of the Philippines Los Baños, College, Laguna, Philippines

## Abstract

A draft genome sequence of a *Streptomyces* sp. isolated from mangrove soil in Cebu, Philippines, is described here. This isolate produced compounds with contact insecticidal activity against important corn pests. The genome contains 7,479,793 bp (in 27 scaffolds), 6,297 predicted genes, and 29 secondary metabolite biosynthetic gene clusters.

## GENOME ANNOUNCEMENT

*Streptomyces* is a soil bacterium known for its production of secondary metabolites, such as antibiotics. Certain *Streptomyces* species also produce polyketide insecticidal compounds, such as avermectin (1), doramectin congeners (2), nanchangmycin, meilingmycin (3), and spinosyn (4, 5), which have been reported to be very active against insects and are nontoxic to mammals and plants. Here, we present the genome sequence of *Streptomyces* sp. strain PCS3-D2, isolated from mangrove soil in Cebu, Philippines, which exhibits insecticidal properties against important pests of corn, such as Asian corn borer (*Ostrinia furnacalis*), earworm (*Helicoverpa armigera*), and cutworm (*Spodoptera litura*).

*De novo* shotgun sequencing was done using Roche Genome Sequencer FLX. A shotgun library and 3-kb mate-pair library were obtained according to the manufacturer’s protocols. The sequence reads were assembled using the GS *de novo* Assembler (version 2.8). The resulting DNA scaffolds were further analyzed using Rapid Annotations using Subsystems Technology (RAST) (6) and the Microbial Genome Annotation Pipeline (MiGAP) (7) for the annotation of rRNA and tRNA. The NCBI Prokaryotic Genome Automatic Annotation Pipeline (PGAAP) was used for gene annotation in preparation for submission to Genbank (http://www.ncbi.nlm.nih.gov/genomes/static/Pipeline.html). The G+C content was analyzed using Seqool (http://www.biossc.de/seqool/index.html).

The draft genome sequence of *Streptomyces* sp. PCS3-D2 (P1) was estimated to be 7,479,793 bp, representing 30× coverage. The assembled genome consists of 27 scaffolds, comprising 138 large contigs. The sizes of the largest scaffold and contig are 1,713,418 and 687,828 bp, respectively. The genome has a G+C content of 72.15%. RAST and MiGAP gene annotation revealed 70 tRNAs representing all 20 standard amino acids and 3 rRNAs (1 5S rRNA, 1 16S rRNA, and 1 23S rRNA). A total of 6,297 coding sequences (CDS), 118 pseudogenes, 12 noncoding RNAs (ncRNAs), and 75 frameshifted genes were predicted using PGAAP.

antiSMASH (8) was used to determine the presence of biosynthetic gene clusters, particularly polyketide synthase (PKS) type I and II and nonribosomal peptide synthase (NRPS) genes, in the *Streptomyces* PCS3-D2 genome sequence. Based on the results, antiSMASH predicted 29 gene clusters, which include genes for type I, II, and III polyketide synthases, NRPS, siderophores, terpenes, butyrolactones, lantibiotics, melanin, and ectoine. These gene clusters may be novel to *Streptomyces* sp. PCS3-D2 and might be responsible for the production of novel insecticidal compounds.

### Nucleotide sequence accession numbers.

This whole-genome shotgun project has been deposited at DDBJ/EMBL/GenBank under the accession no. JDUZ00000000. The version described in this paper is version JDUZ01000000.

## References

[1] Yong JH Byeon WH 2005 Alternative production of avermectin components in *Streptomyces avermitilis* by gene replacement. J. Microbiol. 43:277–284 15995647

[2] Wang XJ Zhang J Wang JD Huang SX Chen YH Liu CX Xiang WS 2011 Four new doramectin congeners with acaricidal and insecticidal activity from *Streptomyces avermitilis* NEAU1069. Chem. Biodivers. 8:2117–2125. 10.1002/cbdv.201000295 22083924

[3] Suny ZX Liu J Bao K Zhang G Tu G Kieser T Deng Z Deng Z 2002 ‘Streptomyces nanchangensis,’ a producer of the insecticidal polyether antibiotic nanchangmycin and the antiparasitic macrolide meilingmycin, contains multiple polyketide gene clusters. Microbiology 148:361–371 11832500 10.1099/00221287-148-2-361

[4] Kim HJ White-Phillip JA Ogasawara Y Shin N Isiorho EA Liu HW 2010 Biosynthesis of spinosyn in *Saccharopolyspora spinosa*: synthesis of permethylated rhamnose and characterization of the functions of SpnH, SpnI, and SpnK. J. Am. Chem. Soc. 132:2901–2903. 10.1021/ja910223x 20158237 PMC2832084

[5] Pan Y Yang X Li J Zhang R Hu Y Zhou Y Wang J Zhu B 2011 Genome sequence of the spinosyns-producing bacterium *Saccharopolyspora spinosa* NRRL 18395. J. Bacteriol. 193:3150–3151. 10.1128/JB.00344-11 21478350 PMC3133185

[6] Aziz RK Bartels D Best AA DeJongh M Disz T Edwards RA Formsma K Gerdes S Glass EM Kubal M Meyer F Olsen GJ Olson R Osterman AL Overbeek RA McNeil LK Paarmann D Paczian T Parrello B Pusch GD Reich C Stevens R Vassieva O Vonstein V Wilke A Zagnitko O 2008 The RAST server: Rapid Annotations using Subsystems Technology. BMC Genomics 9:75. 10.1186/1471-2164-9-75 18261238 PMC2265698

[7] Sugawara H Ohyama A Mori H Kurokawaw K 2009 Microbial genome annotation pipeline (MiGAP) for diverse users, poster S001-1-2. 20th Int. Conf. Genome Informatics (GIW2009), Yokohama, Japan .

[8] Medema MH Blin K Cimermancic P de Jager VD Zakrzewski P Fischbach MA Weber T Takano E Breitling R 2011 antiSMASH: rapid identification, annotation and analysis of secondary metabolite biosynthesis gene clusters in bacterial and fungal genome sequences. Nucleic Acids Res. 39(Suppl 2)**:** W339–W346. 10.1093/nar/gkr466 21672958 PMC3125804

